# Supplementary material for: Regional differences in an established population of invasive Indo-Pacific lionfish (Pterois volitans and P. miles) in south Florida
Source: PeerJ. 2018 Oct 10;6:e5700. doi: 10.7717/peerj.5700 (PMC6186158; doi:10.7717/peerj.5700)
Supplement: Table S1 [file peerj-06-5700-s002.pdf]

Density (ind ha^{-1})	Error	Study Region	Native or Invaded	Years	Lionfish Species	Survey method	# Sites	# Samples	# Lionfish	Reference #
393.3	144.4 (SD)	Bahamas	Invaded	2008	P. volitans	50 x 10 m BT	3	12		29
101.7	103 (SD)	Bahamas	Invaded	2008	P. volitans	50 x 10 m BT	13	75	239	28
13	18 (SD)	Bahamas (San Salvador)	Invaded	2009	P. volitans	50 x 10 m BT	18	54		27
160		Belize	Invaded	2012	P. volitans	50 x 10 m BT	71			26
11.1	4.2 (SE)	Belize	Invaded	2013	P. volitans	50 x 10 m BT	16			56
30		Bonaire (fished)	Invaded	2011	P. volitans	50 x 4 m BT	264		264	65
66		Bonaire (unfished)	Invaded	2011	P. volitans	50 x 4 m BT	156		151	65
440		Caribbean	Invaded	2009-2012	P. volitans	30 x 2m BT & 50 x 10m BT				26
2.88	0.9 (SE)	Colombia	Invaded	2012	P. volitans	50 x 5 m BT		282		66
92	130 (SD)	Costa Rica	Invaded	2011	P. volitans	15-43 x 5 m BT	4	26		67
310		Cuba	Invaded	2013	P. volitans	50 x 2 m BT	6	36		68
160	180 (SD)	Cuba	Invaded	2012-2013	P. volitans	50 X 2m BT	5			69
150		Cuba (Jardines de la Reina)	Invaded	2011	P. volitans	30 x 2 m BT & 50 x 10m BT				26
127		Curacao	Invaded	2011	P. volitans	50 x 4 m BT	132		147	65
3.5	0.9 (SE)	Guam	Native	2010	P. volitans	500 x 10 m BT	23		127	60
3.6		Indian Ocean	Native		Pterois spp.	25-50 x 2-5 m BT		389	61	34
39.51	5.6 (SE)	Jamaica	Invaded	2012-2014	P. volitans	25 x 5 m BT	3	468		70
32.58	4.89 (SE)	Jamaica	Invaded	2012-2014	P. volitans	25 x 5 m BT				70
21.29	3.47 (SE)	Jamaica	Invaded	2012-2014	P. volitans	25 x 5 m BT				70
25.1	45.7 (SD)	Kenya	Native		P. miles	50 x 10 m BT	7	23	70	28
162.5		Little Cayman Island	Invaded	2013	P. volitans	50 x 4 m BT	84			33
34		Little Cayman Island	Invaded	2013	P. volitans	50 x 4 m BT	84			33
21.5		Little Cayman Island	Invaded	2013	P. volitans	50 x 4 m BT	84			33
118	24 (SE)	Martinique	Invaded	2013	P. volitans	50 x 50 m quadrat	5		6848	71
14.7	0.8 (SE)	Martinique	Invaded	2012	P. volitans	50 x 5 m BT	11	39		71
21.2	5.1 (SD)	North Carolina	Invaded	2004	P. volitans	50-100 m x visiblity BTs	17	17		30
84.6	15 (SE)	North Carolina (15-37m)	Invaded	2006-2010	P. volitans	50 x 10 m BT				31
176.4	24 (SE)	North Carolina (38-46m)	Invaded	2006-2010	P. volitans	50 x 10 m BT				31
49		Northern Gulf of Mexico (natural reef)	Invaded	2009-2013	P. volitans	25 x 5m BT, 15 m cylinder	38		934	16
2.2		Palau	Native	2009	P. volitans	rotonone	28			72
300		Panama	Invaded	2013	P. volitans	30 x 3 m BT	3	12		73
21.9	6.5 (SE)	Philippines	Native	2010	P. volitans	500 x 10 m BT	24		397	60
26.4		Red Sea	Native		P. miles	50 x 10 m BT	6	98		74
0.27		Throughout Pacfic Ocean	Native		Pterois spp.	25-50 x 2-5 m BT	110	10204	64	34
16.79		Turks and Caicos (South Caicos)	Invaded	2013	P. volitans	transects, dist. based samp.		37	299	75
121	164 SD	Venezuela	Invaded	2013	P. volitans					76
30	83.5 SD	Venezuela	Invaded	2011	P. volitans	30 x 2 m BT	11	22		76
25.83	66.51 (SD)	Venezuela	Invaded	2014	P. volitans	30 x 4 m BT	5	19	62	77
